# LONG TERM CARE FEASIBILITY OF NURSING HOME LVNS USING AN ALGORITHM-GUIDED ASSESSMENT SUPPORT TOOL

**DOI:** 10.1093/geroni/igz038.1873

**Published:** 2019-11-08

**Authors:** Alyce S Ashcraft, Donna C Owen, Kyle Johnson, Huaxin Song

**Affiliations:** Texas Tech University Health Sciences Center, Lubbock, Texas, United States

## Abstract

Licensed Vocational Nurses (LVNs) are the frontline staff communicating with off-site physicians to manage new or unexpected symptoms in nursing home (NH) residents. Care decisions following physician-LVN conversation impact Alzheimer’s disease (AD) resident quality of care. LVN difficulties using standardized communication forms (e.g. SBAR) spurred creation of an alternative not requiring high levels of RN supervision. The aim of this study was to examine the feasibility of an algorithm guided assessment support tool (AGAST) to help LVNs collect and cluster information using a simulated clinical scenario. A descriptive, participant observation design (video-taped) was used with ten practicing LVNs. AGAST was implemented in the Simulation Center in a clinical scenario of a NH resident with AD experiencing a urinary tract infection. The algorithm was placed within an online survey and participants were directed through a series of sequenced question and answer choices to identify and collect information pertinent to describing a change in resident condition. Duration of videos, description of AGAST-linked assessment behaviors, and content analysis of post-intervention transcripts were reported. AGAST prompts were seen with LVN physical assessment behavior and followed by questions about relevant day-to-day activity/level of performance. AGAST mean completion duration was 11.20 (SD=4.67) minutes. AGAST was rated as easy or very easy (90%). Major concerns included small font size on lab screens, insufficient open text space to add more information, and direct computer input. AGAST was a feasible alternative for assessment by LVNs. Further testing is needed in a variety of conditions.

